# A rare case of mosaic Klinefelter syndrome in a 45-year-old man leading to successful live birth through ejaculated spermatozoa: a case report and literature review

**DOI:** 10.3389/fgene.2023.1242180

**Published:** 2023-09-13

**Authors:** Hossam Elzeiny, Franca Agresta, John Stevens, David K. Gardner

**Affiliations:** ^1^ Melbourne IVF, Melbourne, VIC, Australia; ^2^ Royal Women’s Hospital, Melbourne, VIC, Australia; ^3^ School of Bioscience, University of Melbourne, Parkville, VIC, Australia

**Keywords:** mosaic Klinefelter syndrome, ejaculated human spermatozoa, infertility, severe oligozoospermia, intracytoplasmic sperm injection

## Abstract

**Background:** Men diagnosed with Klinefelter syndrome (KS) commonly exhibit non-obstructive azoospermia or rarely having sperm in their ejaculate, rendering them traditionally considered sterile prior to the introduction of intracytoplasmic sperm injection (ICSI). The presence of mosaic KS may mask the classical phenotype, resulting in underdiagnosis throughout their lifetime. Surgical sperm retrieval through Microdissection Testicular Sperm Extraction (Micro-TESE) combined with ICSI has become the gold standard approach, maximizing reproductive outcomes in these individuals. However, it is noteworthy that approximately 7% of men with KS may exhibit sperm in their ejaculate, providing an opportunity for them to achieve biological parenthood through ICSI.

**Case Presentation:** In this report, we present an exceptional case of a 45-year-old man with Mosaic KS and severe oligozoospermia who successfully achieved pregnancy utilizing ICSI with freshly ejaculated sperm. Remarkably, this case represents the oldest recorded instance of a man with Klinefelter syndrome fathering his own biological child using sperm derived from fresh ejaculate.

**Conclusion:** Although this case is exceedingly rare, it underscores the critical importance of exhausting all possibilities to facilitate biological parenthood in men with KS before considering alternative options such as sperm donation or adoption. By recognizing the potential for successful conception using ejaculated sperm in this population, we can provide individuals with mosaic KS the opportunity to fulfill their desire for biological offspring.

## Introduction

Klinefelter syndrome (KS), initially described by Dr. Harry Klinefelter in 1942, is characterized by bilateral gynecomastia, small testes, aspermatogenesis, reduced Leydig cell function, and elevated follicle-stimulating hormone (FSH) levels (Klinefelter, 1942). In 1959, the genetic origin of KS was elucidated (Jacobs and Strong, 1959). KS is the most prevalent genetic cause of male infertility, typically presenting with non-obstructive azoospermia (Groth et al., 2013). It is also the most commonly observed sex chromosomal abnormality, affecting an estimated frequency of 1 in 500 to 1 in 1,000 men (Abramsky and Chapple, 1997). The classic form of KS, accounting for 80%–90% of cases, is characterized by a 47, XXY karyotype, resulting from sex chromosome aneuploidy. The remaining 10%–20% of cases involve more complex aneuploidies (e.g., 48, XXXY or 48,XXYY), structurally abnormal X chromosomes (e.g., 47,iXq,Y), or mosaicisms (e.g., 47,XXY/46,XY) (Bonomi et al., 2017).

Hypogonadism is a common symptom in KS regardless of genotype (Zitzmann and Rohayem, 2020). Non-mosaic KS individuals tend to exhibit more severe symptoms compared to those with mosaic KS (Paduch et al., 2008; Samplaski et al., 2014). Additionally, men with mosaic KS are generally believed to have higher androgen levels compared to their non-mosaic counterparts (Lanfranco et al., 2004). However, a subset of men with mosaic KS may still display the classic non-mosaic phenotype to some extent (Abdel-Razic et al., 2012). High-grade sex chromosomal aneuploidies (e.g., 48,XXXY, 49,XXXXY, or 48,XXYY) are considered “KS variants” but are associated with severe psychomotor developmental issues, distinguishing them as clinically distinct entities (Lanfranco et al., 2004; Gravholt et al., 2018).

Azoospermia, is a consistent finding in KS, with only approximately 7% of KS males exhibiting detectable spermatozoa in their semen (Aksglaede et al., 2006; Samplaski et al., 2014; Rohayem et al., 2016). Prior to the advent of assisted reproductive techniques, individuals with KS were deemed sterile. Over 95% of individuals were unable to conceive naturally, leading many couples to resort to use of donor sperm or adoption for parenthood (Bojesen et al., 2011; Nahata et al., 2013). However, the discovery of focal areas of spermatogenesis within the testes has positioned testicular sperm extraction (TESE) as a crucial treatment option for sperm retrieval and future reproductive endeavours (Achermann et al., 2021).

The wide phenotypic spectrum of KS contributes to underdiagnosis or delayed diagnosis Notably, KS is frequently diagnosed when individuals seek medical attention for infertility investigations. Consequently, the average age of diagnosis is in the mid-30s, and it is estimated that only 25%–50% of individuals with KS receive a diagnosis (Bojesen et al., 2003; Herlihy and McLachlan, 2015).

The case presented herein focuses on a unique scenario involving a 45-year-old man with mosaic KS and severe oligozoospermia who achieved successful pregnancy through intracytoplasmic sperm injection (ICSI) using ejaculated fresh sperm. This is the first report of the oldest man with mosaic KS to father his offspring using ejaculated spermatozoa.

By highlighting this exceptional case, we aim to increase awareness among healthcare professionals about the potential for successful assisted reproduction in older men with KS. It is crucial to recognize that advanced age in KS individuals should not be a deterrent when considering assisted reproductive technology as an option for achieving biological parenthood.

Approval to publish this case study was granted from the local research committee and the local ethical board (ethics approval no. 84/21-MIVF). Permission to publish was also granted and written consent was obtained from the family.

## Case description

A 45-year-old male and his 32-year-old female partner presented with a 3-year history of primary infertility. Semen analysis of the male partner revealed severe oligozoospermia on two occasions. He had a history of smoking ten cigarettes per day and was undergoing treatment with Sertraline for depression. The patient had no significant family, surgical, or social history.

Physical examination of the male partner showed no abnormalities, with a height of 173 cm and a weight of 74 kg. He exhibited appropriate virilization and had no signs of gynecomastia. Local genital examination revealed normal consistency and small-volume testes, each measuring 6 mL using an orchidometer. The epididymis appeared normal bilaterally, and examination of the spermatic cord revealed intact vas deferens without evidence of clinical varicocele.

Given the severity of oligozoospermia, the patient was advised to cryopreserve his sperm as a precautionary measure for future intracytoplasmic sperm injection (ICSI) procedures. Subsequent semen analyses were performed according to the World Health Organization 2010 guidelines (World Health Organization, 2010), indicating a semen volume of 5 mL, a concentration of 3.7 X10^6^/ml, 42% forward progressive motility, and 0% normal morphology.

To investigate the cause of severe oligozoospermia, the patient underwent chromosomal analysis, Y chromosome microdeletion testing, and hormonal profiling; Karyotyping by G- and C-banding was performed and revealed Mosaic Klinefelter Syndrome, the resolution of G-banding was no less than 550 bands (for details, see ISCN 2020 (McGowan-Jordan et al., 2020).

Initial Karyotype was performed using 20 peripheral blood cells to diagnose KS and to further examine the mosaic status,a second Karyotype examining 100 cells performed showing 46, XY (9 cells) and 47, XXY (91 cells). No Y chromosome microdeletion was detected. Hormonal profiling revealed a morning serum total testosterone level of 14.6 nmol/L, sex hormone-binding globulin (SHBG) level of 47 nmol/L, estradiol (E2) level of 89 pmol/L, follicle-stimulating hormone (FSH) level of 12.5 U/L, and luteinizing hormone (LH) level of 9.9 IU/L.

The female partner had a regular menstrual cycle and reported mild dysmenorrhea but no dyspareunia. She had no history of pelvic inflammatory disease or sexually transmitted infections. Pelvic ultrasound showed no uterine pathology, and the ovaries displayed no cysts, with a total antral follicle count of 13 (8 in the right ovary and 5 in the left ovary). Her Anti-Mullerian Hormone (AMH) level was 8.1 pmol/L (1.13 ng/mL).

Genetic counselling was offered to the couple, but they declined termination of pregnancy if KS was detected. Instead, they opted for prenatal non-invasive genetic screening.

In September 2020, a standard antagonist protocol was initiated, starting from cycle day 3, by administering a daily dosage of 225 IU of recombinant FSH (Gonal F, Merck Serono, Switzerland) for 9 days. On cycle day 8, a gonadotropin-releasing hormone (GnRH) antagonist (Ganirelix 250 mcg, MSD, Macquarie Park, Australia) was introduced to prevent premature ovulation. Follicle development was monitored by transvaginal ultrasound. Once three follicles measuring ≥17 mm in diameter were detected, hCG (Ovidrel 250 mcg; Merck, Macquarie Park, Australia) was administered to trigger ovulation and induce final oocyte maturation. Oocyte retrieval was planned 37 h after trigger using a 17-gauge/35 cm single-lumen needle (Cook Medical, Eight Mile Plains Queensland, Australia) through transvaginal follicle aspiration under ultrasound guidance. Four mature oocytes were retrieved. Simultaneously, a fresh semen sample was obtained from the male partner. A single motile sperm was selected and injected into each of the four metaphase II oocytes.

Results: Three oocytes were fertilized normally, while one embryo arrested. The remaining two embryos developed into blastocysts by day 5 and 6 post-fertilization. The first blastocyst, graded as 3BB according to the Gardner scoring system (Gardner and Schoolcraft, 1999), was suitable for fresh embryo transfer. The second blastocyst, graded as 4AB, was cryopreserved on day 6 post-fertilization for future use. The embryo transfer procedure was performed under abdominal ultrasound guidance using the Cook Sydney IVF Embryo Transfer Set (Cook Medical, United States of America) catheter, and the blastocyst was transferred in approximately 20 μL of Embryo Glue (Vitrolife). Luteal phase support was initiated 2 days after oocyte retrieval using progesterone gel (Crinone 90 mg, Merck, Macquarie Park, Australia).

The patient’s β-hCG level was positive (159 mIU/mL) 9 days after embryo transfer, confirming clinical pregnancy. At 6 weeks and 4 days of gestation, a single intrauterine gestational sac with fetal heartbeat was documented via transvaginal ultrasound, confirming a viable pregnancy. Prenatal genetic screening showed no aneuploidy. The patient successfully delivered a live female offspring weighing 3050 g via caesarean section (due to failure to progress) at 40 weeks and 3 days of gestation in June 2021. The newborn showed no apparent abnormalities.

## Discussion

This case highlights the successful utilization of ICSI in a 45-year-old male with mosaic KS to achieve a viable pregnancy using his own ejaculated spermatozoa. The advanced age of the patient further adds to the uniqueness of this case, positioning him as the oldest man reported to father his own biological child in the setting of KS. Natural conception in men with KS is exceedingly rare, however the first reported case claimed paternity was through blood-group matching (Warburg, 1963). Since then, a further three cases of proven paternity have been reported (Laron et al., 1982; Terzoli et al., 1992; Li, 2011). Following the introduction of ICSI in 1992, the first live birth for a 29-year-old man with KS with spermatozoa in his ejaculate using ICSI was reported by (Bourne et al., 1997).

In our case, the diagnosis of KS was initially made during infertility investigations, consistent with previous reports indicating that approximately half of KS cases are diagnosed in adulthood due to fertility-related issues (Bojesen et al., 2003). The patient exhibited a normal phenotype with mildly elevated follicle-stimulating hormone (FSH) levels and a low borderline testosterone level, accompanied by severe oligozoospermia, which can be attributed to the mosaic nature of his genotype. It is important to note that some men with mosaic KS may present with phenotypic characteristics that partially resemble the classic non-mosaic phenotype, and azoospermia has been reported in approximately 75% of individuals with mosaic KS (Plotton et al., 2014).

The intricate relationship between genotype and phenotype in Klinefelter syndrome (KS) remains an ongoing challenge to elucidate comprehensively. While the impact of a specific gene, SHOX, has been identified in contributing to certain phenotypic aspects of KS, the broader genotype-phenotype correlation remains enigmatic. Available literature underscores the notion that a singular genetic mechanism does not solely account for the wide-ranging phenotypic spectrum and variability seen in KS. Rather, it appears that a confluence of factors, including genetic, such as number of CAG repeats in the androgen receptor, parent of origin of the extra X chromosome or skewed inactivation of one X chromosome, additionally epigenetic, hormonal, environmental, and individual influences, likely interact to shape the observed variations (Skakkebaek et al., 2020).

The presence of an additional X chromosome and the associated gene dosage effect, along with hypogonadism, can partially explain certain phenotypic features observed in KS (Skakkebaek et al., 1969).

The cytogenetic analysis of our case demonstrated clearly a 9% mosaicism. However, it is crucial to recognize that the karyotype observed in lymphocytes may not directly correlate with sperm production or accurately represent the karyotype of the testicular germ cell line. Therefore, caution must be exercised when using karyotype as a predictive factor for sperm retrieval in cases of non-mosaic KS (Westlander et al., 2001).

Furthermore, men with KS may exhibit tissue-specific mosaicism, indicating that relying solely on peripheral karyotyping may fail to capture the mosaic status of the condition (Lanfranco et al., 2004). While peripheral leukocyte karyotype analysis may reveal a classic non-mosaic KS, the testicular tissue may harbor a mosaic KS karyotype (Garcia-Quevedo et al., 2011). This discrepancy poses a challenge in accurately determining the true frequency of mosaicism in KS men.

Different predictive markers have been explored for successful sperm retrieval in KS, including mosaic state, testosterone level, and age. While some studies suggest that age has no effect on predicting sperm retrieval in KS men (Garolla et al., 2018), others have concluded that age is the only predictive factor for successful sperm retrieval (Okada et al., 2005; Emre Bakircioglu et al., 2006; Ferhi et al., 2009; Ramasamy et al., 2009; Sabbaghian et al., 2014). Specifically, men with KS over the age of 35 have been found to have the lowest sperm retrieval rate (Okada et al., 2005; Emre Bakircioglu et al., 2006; Ferhi et al., 2009; Ramasamy et al., 2009). However, the successful pregnancy and delivery in our case challenge the notion that older age precludes men with mosaic KS from pursuing assisted reproductive technologies.

To date, a total of 10 couples with KS male partners who have sperm in their ejaculate have achieved live births, resulting in 15 children (including 5 singletons and 5 sets of twins). The male partners’ ages ranged from 29 to 35 years old (Table 1). In all these cases, assisted reproductive technology using ICSI was necessary for conception. Seven couples had double embryo transfer and two has three embryos transferred, all has cleavage stage transfers except two who had their embryos transferred at the blastocyst stage following analysis by FISH on cleavage stage embryos. Our case is in line with the current practice of elective single blastocyst transfer and achieved singleton delivery. The need for genetic counselling in couples with KS males is still a matter of debate. However, it is important to initiate a discussion regarding the option of non-invasive prenatal testing (NIPT) or prenatal diagnosis with the couple (Mazloom et al., 2013).

**TABLE 1:** live birth outcome of Klinefelter syndrome men who have spermatozoa in their ejaculate.

Study	Age	Mosaic state	Sperm utilised	Sperm concentration	Fertilisation rate	Genetic testing	ET day	ET #	Outcome
	Years								
Bourne et al. (1997)	29	NM	Frozen	Zero	10/13(77%)	Declined	D2	2	XX/XY
Bielanska et al. (2000)	35	M	N/A	0.1x10^6^/ml	15/19(79%)	Offered	N/A	3	XX/XX
Crüger et al. (2001)	28	NM	Fresh	N/A	4/4(100%)	Declined	D2	2	XX
Staessen et al. (2003)	N/A	N/A	Fresh	N/A	N/A	D3 FISH	D5	2	Singleton
Tachdijan et al. (2003)	27	NM	Fresh	Rare	5/9(56%)	Not performed	N/A	2	XX/XY
Komori et al. (2004)	31	NM	Fresh	0.35x10^6^/ml	3/3(1%)	N/A	N/A	2	XX
Akashi et al. (2005)	35	M	Fresh	0.4x10^6^/ml	5/6(83%)	Sperm FISH	D2	3	XX
Zhang et al. (2013)	24	NM	Fresh	0.7x10^6^/ml	11/11(100%)	N/A	D3	3	XX/XY
Gurbuz et al. (2015)	34	NM	Fresh	2x10^6^/ml	10/11(91%)	D3 FISH	D5	3	XX/XY
Xu et al. (2022)	31	NM	Fresh	1.7x10^6^/ml	4/7(57%)	Declined	D3	1	XY

**Appreciations;** N/A not applicable, NM, Non-Mosaic, M Mosaic, FISH, fluorescence *in situ* hybridization.

Although NIPT has limitations in detecting sex-chromosome aneuploidies, it has become more accessible to patients. A large study evaluating the performance of NIPT in detecting sex chromosome aneuploidy reported a positive predictive value of 66.67% for 47, XXY (Lu et al., 2021). The lower prevalence of KS, combined with potential false-positive results due to maternal mosaicism, contributes to the lower predictive value. The value of preimplantation genetic testing for aneuploidy (PGT-A) in couples with KS individuals remains debatable. The incidence of aneuploidy in offspring from men with classic KS is only 1%, and there are no significant differences in pregnancy rates or miscarriage rates compared to men with non-obstructive azoospermia (Bonduelle et al., 2002; Plotton et al., 2014). Therefore, the endorsement of PGT-A or prenatal genetic analyses for these couples is still a topic of discussion (Zitzmann et al., 2021).

## Conclusion

This was a rare case of a man with mosaic Klinefelter syndrome who presented with severe oligospermia and a successful live birth was achieved using assisted reproductive technology, the classical presentation of KS is azoospermia and surgical sperm retrieval using MicroTESE is required with variable success rate. We hope that this case sheds light on the awareness of the phenotypic variation of KS with respect to age and contributes to the existing literature.

## Data Availability

The raw data supporting the conclusion of this article will be made available by the authors, without undue reservation.

## References

[B1] Abdel-Razic M. M. Abdel-Hamid I. A. Elsobky E. El-Dahtory F. (2012). Further evidence of the clinical, hormonal, and genetic heterogeneity of klinefelter syndrome: A study of 216 infertile Egyptian patients. J. Androl. 33 (3), 441–448. 10.2164/jandrol.110.011536 21757513

[B2] Abramsky L. Chapple J. (1997). 47,XXY (klinefelter syndrome) and 47,XYY: estimated rates of and indication for postnatal diagnosis with implications for prenatal counselling. Prenat. Diagn. 17 (4), 363–368. 10.1002/(sici)1097-0223(199704)17:4<363::aid-pd79>3.0.co;2-o 9160389

[B3] Achermann A. P. P. Pereira T. A. Esteves S. C. (2021). Microdissection testicular sperm extraction (micro-TESE) in men with infertility due to nonobstructive azoospermia: summary of current literature. Int. Urology Nephrol. 53 (11), 2193–2210. 10.1007/s11255-021-02979-4 34410586

[B4] Akashi T. Fuse H. Kojima Y. Hayashi M. Honda S. (2005). Birth after intracytoplasmic sperm injection of ejaculated spermatozoa from a man with mosaic Klinefelter's syndrome. Asian J. Androl. 7 (2), 217–220. 10.1111/j.1745-7262.2005.00023.x 15897980

[B5] Aksglaede L. Wikström A. M. Rajpert-De Meyts E. Dunkel L. Skakkebaek N. E. Juul A. (2006). Natural history of seminiferous tubule degeneration in Klinefelter syndrome. Hum. Reprod. update 12 (1), 39–48. 10.1093/humupd/dmi039 16172111

[B6] Bielanska M. Tan S. L. Ao A. (2000). Fluorescence *in-situ* hybridization of sex chromosomes in spermatozoa and spare preimplantation embryos of a Klinefelter 46,XY/47,XXY male. Hum. Reprod. Oxf. Engl. 15 (2), 440–444. 10.1093/humrep/15.2.440 10655319

[B7] Bojesen A. Juul S. Gravholt C. H. (2003). Prenatal and postnatal prevalence of klinefelter syndrome: A national registry study. J. Clin. Endocrinol. Metab. 88 (2), 622–626. 10.1210/jc.2002-021491 12574191

[B8] Bojesen A. Stochholm K. Juul S. Gravholt C. H. (2011). Socioeconomic trajectories affect mortality in Klinefelter syndrome. J. Clin. Endocrinol. Metab. 96 (7), 2098–2104. 10.1210/jc.2011-0367 21565794

[B9] Bonduelle M. Liebaers I. Deketelaere V. Derde M. P. Camus M. Devroey P. (2002). Neonatal data on a cohort of 2889 infants born after ICSI (1991-1999) and of 2995 infants born after IVF (1983-1999). Hum. Reprod. Oxf. Engl. 17 (3), 671–694. 10.1093/humrep/17.3.671 11870121

[B10] Bonomi M. Rochira V. Pasquali D. Balercia G. Jannini E. A. Ferlin A. (2017). Klinefelter syndrome (KS): genetics, clinical phenotype and hypogonadism. J. Endocrinol. Invest. 40 (2), 123–134. 10.1007/s40618-016-0541-6 27644703 PMC5269463

[B11] Bourne H. Stern K. Clarke G. Pertile M. Speirs A. Baker H. W. (1997). Delivery of normal twins following the intracytoplasmic injection of spermatozoa from a patient with 47,XXY Klinefelter's syndrome. Hum. Reprod. Oxf. Engl. 12 (11), 2447–2450. 10.1093/humrep/12.11.2447 9436682

[B12] Crüger D. Toft B. Agerholm I. Fedder J. Hald F. Bruun-Petersen G. (2001). Birth of a healthy girl after ICSI with ejaculated spermatozoa from a man with non-mosaic Klinefelter's syndrome. Hum. Reprod. Oxf. Engl. 16 (9), 1909–1911. 10.1093/humrep/16.9.1909 11527897

[B13] Emre Bakircioglu M. Erden H. F. Kaplancan T. Ciray N. Bener F. Bahceci M. (2006). Aging may adversely affect testicular sperm recovery in patients with Klinefelter syndrome. Urology 68 (5), 1082–1086. 10.1016/j.urology.2006.05.028 17095066

[B14] Ferhi K. Avakian R. Griveau J. F. Guille F. (2009). Age as only predictive factor for successful sperm recovery in patients with Klinefelter's syndrome. Andrologia 41 (2), 84–87. 10.1111/j.1439-0272.2008.00875.x 19260843

[B15] Garcia-Quevedo L. Blanco J. Sarrate Z. Català V. Bassas L. Vidal F. (2011). Hidden mosaicism in patients with klinefelter's syndrome: implications for genetic reproductive counselling. Hum. Reprod. Oxf. Engl. 26 (12), 3486–3493. 10.1093/humrep/der351 22016414

[B16] Gardner D. K. Schoolcraft W. B. (1999). Culture and transfer of human blastocysts. Curr. Opin. Obstetrics Gynecol. 11 (3), 307–311. 10.1097/00001703-199906000-00013 10369209

[B17] Garolla A. Selice R. Menegazzo M. Valente U. Zattoni F. Iafrate M. (2018). Novel insights on testicular volume and testosterone replacement therapy in Klinefelter patients undergoing testicular sperm extraction. A retrospective clinical study. Clin. Endocrinol. 88 (5), 711–718. 10.1111/cen.13572 29446828

[B18] Gravholt C. H. Chang S. Wallentin M. Fedder J. Moore P. Skakkebæk A. (2018). Klinefelter syndrome: integrating genetics, neuropsychology, and endocrinology. Endocr. Rev. 39 (4), 389–423. 10.1210/er.2017-00212 29438472

[B19] Groth K. A. Skakkebæk A. Høst C. Gravholt C. H. Bojesen A. (2013). Clinical review: klinefelter syndrome--a clinical update. J. Clin. Endocrinol. Metab. 98 (1), 20–30. 10.1210/jc.2012-2382 23118429

[B20] Gurbuz A. S. Salvarci A. Ozcimen N. Zamani A. G. (2015). Early morphokinetic monitoring of embryos after intracytoplasmic sperm injection with fresh ejaculate sperm in nonmosaic klinefelter syndrome: A different presentation. Case Rep. Genet. 2015, 827656. 10.1155/2015/827656 26843994 PMC4710914

[B21] Herlihy A. S. McLachlan R. I. (2015). Screening for klinefelter syndrome. Curr. Opin. Endocrinol. Diabetes, Obes. 22 (3), 224–229. 10.1097/MED.0000000000000154 25871960

[B22] Jacobs P. A. Strong J. A. (1959). A case of human intersexuality having a possible XXY sex-determining mechanism. Nature 183 (4657), 302–303. 10.1038/183302a0 13632697

[B23] Klinefelter H. F. Reifenstein E. C. Albright F. (1942). Syndrome characterized by gynecomastia, aspermatogenesis without A-leydigism, and increased excretion of follicle-stimulating Hormone1. Clin. Endocrinol. (Oxf) 2 (2), 615–627. 10.1210/jcem-2-11-615

[B24] Komori S. Horiuchi I. Hamada Y. Hasegawa A. Kasumi H. Kondoh N. (2004). Birth of healthy neonates after intracytoplasmic injection of ejaculated or testicular spermatozoa from men with nonmosaic klinefelter's syndrome: A report of 2 cases. J. Reproductive Med. 49 (2), 126–130. 15018443

[B25] Lanfranco F. Kamischke A. Zitzmann M. Nieschlag E. (2004). Klinefelter’s syndrome. Lancet 364 (9430), 273–283. 10.1016/S0140-6736(04)16678-6 15262106

[B26] Laron Z. Dickerman Z. Zamir R. Galatzer A. (1982). Paternity in Klinefelter's syndrome--a case report. Archives Androl. 8 (2), 149–151. 10.3109/01485018208987032 6803694

[B27] Li D. Z. (2011). Paternity in Klinefelter syndrome - another case report. J. Endocrinol. Invest. 34 (7), 570. 10.1007/BF03345395 21897109

[B28] Lu X. Wang C. Sun Y. Tang J. Tong K. Zhu J. (2021). Noninvasive prenatal testing for assessing foetal sex chromosome aneuploidy: A retrospective study of 45,773 cases. Mol. Cytogenet. 14 (1), 1. 10.1186/s13039-020-00521-2 33407708 PMC7786464

[B29] Mazloom A. R. Džakula Ž. Oeth P. Wang H. Jensen T. Tynan J. (2013). Noninvasive prenatal detection of sex chromosomal aneuploidies by sequencing circulating cell-free DNA from maternal plasma. Prenat. Diagn. 33 (6), 591–597. 10.1002/pd.4127 23592550

[B30] McGowan-Jordan J. Hastings R. J. Moore S. (Editors) (2020). ISCN 2020— (2020) an international system for human cytogenomic nomenclature (Karger). 10.1159/000516655 34407535

[B31] Nahata L. Rosoklija I. Yu R. N. Cohen L. E. (2013). Klinefelter syndrome: are we missing opportunities for early detection? Clin. Pediatr. 52 (10), 936–941. 10.1177/0009922813493831 23836810

[B32] Okada H. Goda K. Yamamoto Y. Sofikitis N. Miyagawa I. Mio Y. (2005). Age as a limiting factor for successful sperm retrieval in patients with nonmosaic Klinefelter's syndrome. Fertil. Steril. 84 (6), 1662–1664. 10.1016/j.fertnstert.2005.05.053 16359961

[B33] Paduch D. A. Fine R. G. Bolyakov A. Kiper J. (2008). New concepts in Klinefelter syndrome. Curr. Opin. Urol. 18 (6), 621–627. 10.1097/MOU.0b013e32831367c7 18832949

[B34] Plotton I. Brosse A. Cuzin B. Lejeune H. (2014). Klinefelter syndrome and TESE-ICSI. Ann. Endocrinol. 75 (2), 118–125. 10.1016/j.ando.2014.04.004 24786702

[B35] Ramasamy R. Ricci J. A. Palermo G. D. Gosden L. V. Rosenwaks Z. Schlegel P. N. (2009). Successful fertility treatment for Klinefelter's syndrome. J. Urology 182 (3), 1108–1113. 10.1016/j.juro.2009.05.019 19616796

[B36] Rohayem J. Nieschlag E. Zitzmann M. Kliesch S. (2016). Testicular function during puberty and young adulthood in patients with Klinefelter's syndrome with and without spermatozoa in seminal fluid. Andrology 4 (6), 1178–1186. 10.1111/andr.12249 27611179

[B37] Sabbaghian M. Modarresi T. Hosseinifar H. Hosseini J. Farrahi F. Dadkhah F. (2014). Comparison of sperm retrieval and intracytoplasmic sperm injection outcome in patients with and without Klinefelter syndrome. Urology 83 (1), 107–110. 10.1016/j.urology.2013.09.021 24210565

[B38] Samplaski M. K. Lo K. C. Grober E. D. Millar A. Dimitromanolakis A. Jarvi K. A. (2014). Phenotypic differences in mosaic Klinefelter patients as compared with non-mosaic Klinefelter patients. Fertil. Steril. 101 (4), 950–955. 10.1016/j.fertnstert.2013.12.051 24502895

[B39] Skakkebaek A. Viuff M. Nielsen M. M. Gravholt C. H. (2020). Epigenetics and genomics in Klinefelter syndrome. Am. J. Med. Genet. Part C, Seminars Med. Genet. 184 (2), 216–225. 10.1002/ajmg.c.31802 32484281

[B50] Skakkebaek N. E. Philip J. Hammen R. (1969). Meiotic chromosomes in Klinefelter’s syndrome. Nature 221 (5185), 1075–1076. 10.1038/2211075a0 5774408

[B40] Staessen C. Tournaye H. Van Assche E. Michiels A. Van Landuyt L. Devroey P. (2003). PGD in 47, XXY Klinefelter’s syndrome patients. Hum. Reprod. Update 9 (4), 319–330. 10.1093/humupd/dmg029 12926526

[B41] Tachdjian G. Frydman N. Morichon-Delvallez N. Dû A. L. Fanchin R. Vekemans M. (2003). Reproductive genetic counselling in non-mosaic 47,XXY patients: implications for preimplantation or prenatal diagnosis: case report and review. Hum. Reprod. Oxf. Engl. 18 (2), 271–275. 10.1093/humrep/deg070 12571161

[B42] Terzoli G. Lalatta F. Lobbiani A. Simoni G. Colucci G. (1992). Fertility in a 47,XXY patient: assessment of biological paternity by deoxyribonucleic acid fingerprinting. Fertil. Steril. 58 (4), 821–822. 10.1016/s0015-0282(16)55334-5 1426331

[B43] Warburg E. (1963). A fertile patient with Klinefelter's syndrome. Acta Endocrinol. 43, 12–26. 10.1530/acta.0.0430012 13998839

[B44] Westlander G. Ekerhovd E. Granberg S. Hanson L. Hanson C. Bergh C. (2001). Testicular ultrasonography and extended chromosome analysis in men with nonmosaic klinefelter syndrome: A prospective study of possible predictive factors for successful sperm recovery. Fertil. Steril. 75 (6), 1102–1105. 10.1016/s0015-0282(01)01793-9 11384633

[B45] World Health Organization (2010). WHO laboratory manual for the examination of human semen and sperm–cervical mucus interaction. 5th ed. Geneva: World Health Organization.

[B46] Xu W-Q. Yuan Y. Chen Y. Luo T. Chen H-Y. (2022). Birth of a boy after intracytoplasmic sperm injection using ejaculated spermatozoa from a nonmosaic klinefelter syndrome man with normal sperm motility: A case report. Front. Genet. 13, 989701. 10.3389/fgene.2022.989701 36212158 PMC9538340

[B47] Zhang C. Peng H. Hu Y. (2013). Twin pregnancy obtention of patient with nonmosaic klinefelter's syndrome and his wife with chromosome 9 inversion by ICSI treatment. Int. J. Fertil. Steril. 7 (2), 142–146. 24520478 PMC3850336

[B48] Zitzmann M. Aksglaede L. Corona G. Isidori A. M. Juul A. T'Sjoen G. (2021). European academy of andrology guidelines on klinefelter syndrome endorsing organization: european society of endocrinology. Andrology 9 (1), 145–167. 10.1111/andr.12909 32959490

[B49] Zitzmann M. Rohayem J. (2020). Gonadal dysfunction and beyond: clinical challenges in children, adolescents, and adults with 47,XXY klinefelter syndrome. Am. J. Med. Genet. Part C, Seminars Med. Genet. 184 (2), 302–312. 10.1002/ajmg.c.31786 32415901

